# Development of a conceptual framework to scale up co-managed care for older patients with hip fracture in China: a qualitative study

**DOI:** 10.1186/s12913-023-09910-w

**Published:** 2023-08-23

**Authors:** Jing Zhang, Pengpeng Ye, Minghui Yang, Xinbao Wu, Ruth Webster, Rebecca Ivers, Maoyi Tian

**Affiliations:** 1 Clinical Epidemiology Research Centre, Beijing Jishuitan Hospital, Capital Medical University, Beijing, China; 2 National Centre for Orthopaedics, Beijing, China; 3 School of Population Health, University of New South Wales, Sydney, NSW Australia; 4 National Centre for Non-Communicable Disease Control and Prevention, Chinese Centre for Disease Control and Prevention, Beijing, China; 5 Department of Orthopaedics and Traumatology, Beijing Jishuitan Hospital, Capital Medical University, Beijing, China; 6 The George Institute for Global Health, University of New South Wales, Sydney, NSW Australia; 7 School of Public Health, Harbin Medical University, Harbin, China

**Keywords:** Hip fracture, Co-managed care, Conventional care, Multidisciplinary management, Consolidated framework for implementation research

## Abstract

**Background:**

Hip fracture creates a major burden on society due to high mortality, loss of independence and excess medical costs for older people. A multidisciplinary co-managed model of care is widely considered as the best practice for the management of older patients with hip fracture. The study aims to develop a conceptual framework to inform the future scale-up of this model of care through the identification of barriers and enablers that may influence successful uptake.

**Methods:**

This qualitative study was conducted within an interventional study, which aimed to test the effectiveness of co-managed model of care for older patients with hip fracture. Health providers and health administrators from three hospitals were purposively selected and interviewed in-depth. The Consolidated Framework for Implementation Research (CFIR) was used to develop interview guides, collect and analyse data. Inductive and deductive approaches were used to generate enablers or barriers, aligned with the CFIR constructs. All barriers or enablers were inductively summarised to a conceptual framework with essential components to guide the implementation of co-managed model of care in other hospitals.

**Results:**

A total of 13 health providers and 3 health administrators were recruited. The main barriers to co-managed care implementation included perceived complexity of implementation, insufficient international collaboration and incentives, the absence of national guideline support and lack of digital health applications for communication between health providers, insufficient number of health providers and beds, and poor understanding about the effectiveness of this care model. A conceptual framework for future scale-up was then developed, consisting of the following essential components: hospital authority support, enabling environment, adequate number of beds, sufficient and skilled health providers, use of digital health technology, regular quality supervision, evaluation and feedback, and external collaborations.

**Conclusions:**

Despite the complexity of the intervention, the co-managed model of care has the potential to be implemented and promoted in China and in similar settings, although there is a need to demonstrate feasibility in different settings.

**Supplementary Information:**

The online version contains supplementary material available at 10.1186/s12913-023-09910-w.

## Introduction

Hip fracture can be a devastating injury for older adults, particularly those who have existing osteoporosis. Hip fracture can lead to death, loss of independence and/or excess medical costs [1–4]. Globally, the incidence and prevalence of hip fracture patients have increased 92.7% and 113.3% from 1990 to 2019 [5]. This dramatic increase in hip fracture has also been observed amongst the older Chinese population, increasing from 0.7 million cases in 2006 to about 2 million in 2016. Correspondingly, direct medical costs for hip fracture treatment increased about 6-fold from US$60 million in 2012 to US$380 million in 2016, imposing a significant financial burden on patients and health systems in China [2, 6].

A UK published best-practice guideline for hip fracture management, called the “Blue Book”, recommends that older patients with hip fracture should access to orthogeriatric care services [7]. The orthogeriatric care model, essentially co-managed care provided by both orthopaedic surgeons and geriatricians, can accelerate the time to surgery, improve secondary prevention of osteoporosis and falls, as well as reduce patients’ in-hospital and one-year complications after surgery [8–10]. Despite well-established evidence and guidelines, the uptake of this co-managed model of care remains underutilised in China. Instead, the common care model, implemented in most Chinese hospitals, is primarily provided by the orthopaedic surgeon with ad hoc geriatrician consultation [11]. Such fragmented care can lead to delay in surgery and further increase the potential risk of mortality and disability, due to the limited availability of geriatricians [8, 12].

A recently published work that evaluated the effectiveness of this co-managed model of care model amongst Chinese hip fracture patients in Beijing found that this care model had the potential to expedite surgery, improve clinical management and reduce one-year mortality [13]. Co-managed care, using adapted recommendations of the “Blue Book”, was jointly led by the orthopaedic surgeons and geriatricians with involvement of a multidisciplinary team, and was provided to the patients from their arrival at the Emergency Department (ED) until their discharge from the hospital [9], including pre-operative assessment, comorbidity treatment, peri-operative management, post-operative prevention of complications and secondary prevention of osteoporosis and falls. Details of the evaluation of this co-managed model of care have been described elsewhere [13].

Implementation science is an emerging research field to promote the systematic uptake of research findings and other evidence-based practices into routine practice, so as to improve the quality and effectiveness of health services [14]. In order to understand how best to scale up this intervention, it is necessary to understand the factors influencing the implementation of this care model. The implementation science was therefore adopted to design a qualitative study in the hospitals involved in our prior study to identify the barriers and enablers that influenced implementation of co-managed care, and further to develop a conceptual framework to inform the future scale-up of this care model.

## Methods

### Study design and settings

Face-to-face in-depth interviews were conducted to obtain key stakeholders’ perspectives regarding influential factors relevant to delivery of co-managed care for older hip fracture patients. Participants were selected from three heterogeneous acute hospitals from Beijing, China. The three hospitals participated in the previous implementation and evaluation of the co-managed care program. A Hospital is a tier 3 (tertiary, the highest level), leading general hospital (around 2,200 beds) with the orthopaedics and implemented the co-managed model of care as the intervention group of the previous published study. B Hospital is a tier 3 hospital (around 1,250 beds) specialising in geriatrics, while C Hospital is a tier 3, suburban hospital (around 850 beds) focusing on orthopaedics; both delivered orthopaedic surgeon led usual care as the control group of the published study. Consolidated Criteria for Reporting Qualitative Research (COREQ) was applied for reporting the study results (Supplementary 1) [15].

### Study participants

A purposive sampling approach was used to recruit participants consisting of clinicians participating in the management of the older hip fracture patients, including orthopaedic surgeons, geriatricians, anaesthetists, rehabilitation specialists and nurses, and hospital administrators. All potential participants were identified through existing collaborations and recruited by research staff through emails or by phone.

### Analytic framework

The Consolidated Framework for Implementation Research (CFIR) was adopted for the development of interview guides, data synthesis and analysis. The CFIR offers a meta-theoretical framework for identifying and summarising implementation determinants from stakeholders’ perspectives, by synthesising a range of implementation theories in dissemination, innovation, organizational change, implementation, knowledge translation and research uptake [16]. CFIR consists of 5 domains (intervention characteristics, outer setting, inner setting, characteristics of individuals, process), along with 39 constructs (Supplementary 2). The use of the CFIR framework can help identify barriers and enablers that influence the implementation of the co-managed care and also guide planning and evaluation of the care model to bring evidence-based research into practice [17, 18].

### Interview guides and data collection

Semi-structured interview guides were developed, aligned with the CFIR constructs, with specific interview guides developed for the health providers and health administrators respectively. Participants in the intervention group were asked about the challenges they faced during implementation of this care model, while for the control group, participants were asked about the potential challenges if the care model were implemented at their hospitals. All participants were also asked regarding the key determinants for future scale-up of the co-managed model of care.

Face-to-face interviews were conducted from October to December 2020. To build participant trust, and ensure that they were able to share their views freely and confidentially, all interviews were conducted in a private and quiet room by one of the authors (JZ, PhD candidate, male), a native Chinese speaking researcher with extensive qualitative research experience. Written informed consent was obtained from all participants before commencing the interviews. Consent included the permission to be audio-recorded. The sample size was determined by reaching information saturation at interim analysis.

### Data analysis

The audio-recorded interviews were transcribed verbatim. Both inductive and deductive approaches were applied for the qualitative analysis. The codebook was generated with the following steps. First, the lead author (JZ) initially analysed two random transcripts to inductively generate codes; Second, the CFIR constructs were deductively mapped to the emerging codes; Third, using the same method, the second author (PY, male, PhD candidate) analysed the same two transcripts, during which the codebook was refined until an agreement about a hierarchy of conceptual codes and subcodes was reached between the two coders. After that, the two coders (JZ and PY) applied the refined codebook to independently analyse all transcripts. During coding, all discrepancies were resolved by internal discussion in order to increase the intercoder reliability and reach a consensus. The senior authors (RI, RW and MT) were consulted when discrepancies between the coders were not resolved. Aligning with the CFIR constructs, all codes were deductively summarised to themes and subthemes, and were then denoted as enablers or barriers. These enablers or barriers, as the key determinants that influenced the implementation of the co-managed care, were then inductively summarised as a conceptual framework to guide its implementation in other hospitals. Typically, de-identified quotations are included to support the identified enablers and barriers.

NVivo Pro (version 12) qualitative analytical software was used for data analysis. The data were analysed in the Chinese language. All emerging enablers and barriers with quotations were translated into English to delineate the research findings. A back-translation approach was applied to ensure the accuracy of the translated enablers and barriers, and associated quotations.

## Results

A total of 16 participants were recruited from three acute hospitals prior to information saturation being reached. Of these, there were six participants from the co-managed care group, including one orthopaedic surgeon, one geriatrician, one anaesthetist, one rehabilitation specialist, one nurse and one health administrator, while the other ten participants were involved in the conventional care model, consisting of two orthopaedic surgeons, two geriatricians, one anaesthetist, one rehabilitation specialist, two nurses, and two health administrators. The age range of the health providers spanned from 23 to 44 years old, and their length range of employment was from 2 to 17 years, while the age range of the health administrators were from 49 to 56 years old with length of employment spanning from 25 to 32 years.

The average length of the interviews was 42 min, ranging from 31 to 54 min. The identified barriers and facilitators spanned all domains of the CFIR. Enablers and barriers are summarised with associated quotations to support these enablers and barriers in Table 1.

**Table 1:** Themes under CFIR constructs – The influential factors for the implementation of the co-managed model of care

CFIR Domains	Constructs	Enablers	Barriers	Quotations
**Intervention characteristics**	Evidence Strength and Quality	Adapted from evidence-based guidelines of hip fracture		*“……compared to usual care, the co-managed care has been proved it can improve quality of hip fracture care……” (administrator, 0601, co-managed care)*
	Relative Advantage	Offering expedited surgery*		*“……the time to surgery is usually more than 3 days in usual care. For those patients who have comorbidities, internal physicians as a consultant often have a delay for assessments and treatment……” (orthopaedic surgeon, 0103, conventional care)*
	Complexity		More complex than usual care*	*“……I consider that it will be a huge challenge if the co-managed model of care would implement in my hospital, because it is quite hard to coordinate many disciplines during the management……” (administrator, 0602, conventional care)*
	Adaptability		Challenging to be introduced into different settings	*“……I don’t think it is easy for some hospitals with insufficient providers to adopt this care model. For example, geriatricians are lacking in my hospital. They have to overcome that, in terms of replacing geriatricians with other disciplines……” (orthopaedic surgeon, 0101, co-managed care)*
	Design Quality and Packaging		Inconvenient paper-based manual of operation	*“It is inconvenient for me to check the manual of operation because I sometimes forget to take it. An electronic version built in my phone could be better.” (nurse, 0501, co-managed care)*
**Outer setting**	External Policies and Incentives		Lack of guideline support (national level)	*“……We usually provide consultative care for hip fracture patients, and currently no guideline guides us to provide co-managed hip fracture care in China. I often follow the instruction from my supervisor……” (orthopaedic surgeon, 0102, conventional care)*
			Lack of perceived importance of hip fracture management (hospital level)	*“……In China, if hospital president doesn’t want to support the reformation of a care model, nothing can be carried out, because they don’t consider the importance of reorientation of a care model……” (administrator, 0601, co-managed care)*
			Lack of hospital authority’s support (hospital level)*	*“……I consider that it is very hard to establish a co-managed model of care for hip fracture patients in my hospital, because I consider other disciplines, like cardiac, are more important than hip fracture……” (administrator, 0602, conventional care)*
	Peer Pressure	More willingness to improve hip fracture care		*“……We felt the pressure of our colleagues from western countries when we attended international conferences or reading some publications. I think the co-managed care should be understood and promoted in China……” (orthopaedic, 0102, conventional care)*
	Cosmopolitanism		Lack of international and domestic collaborations	*“……Unfortunately, I have no opportunity to collaborate with them (colleagues from high-income countries) ……” (anaesthetist, 0301, co-managed care)*
**Inner setting**	Available Resources	Prioritised pre-operative assessment	Insufficient beds in wards	*“Hospital presidents pushed associated departments to assist the co-managed care, such as blood testing, imaging……” (anaesthetist, 0301, co-managed care)* *“The biggest concern is that we have only 18 beds in the ward, which is insufficient for the increasing number of patients……” (orthopaedic surgeon, 0101, co-managed care)*
	Access to Knowledge and Information	Regularly updated knowledge base		*“We have a regular group discussion to update the knowledge and address problems we encountered. I think it is a really good way to improve the efficacy……” (rehabilitation specialist, 0401, co-managed care)*
	Tension for change		Insufficient geriatricians*	*“The insufficiency of geriatricians needs to be improved immediately if the co-managed care would be implemented……” (administrator, 0603, conventional care)*
	Compatible		Incompatible with previous workflow and routine practice	*“I think basically working in the multidisciplinary team is quite different with my previous routine work. Sometimes I feel a huge pressure and can’t accept……” (nurse, 0501, co-managed care)*
	Networks and Communications	Digital health driven communications between health care providers	The nonspecific app used, incurred potential risk of information leakage	*“……Wechat is the most popular app. I usually use it to send patients’ information to my colleagues. That’s really convenient……” (geriatrician, 0202, conventional care)* *“……but it is a business application, not a specific one for the co-managed care. I am afraid of leaking the private information……” (geriatrician, 0201, co-managed care)*
**Characteristics of individuals**	Knowledge and Beliefs about the Intervention	Having confidence in the success of the intervention		*“We’ve all heard that a significantly positive result was identified in a previous pilot study, that means the co-managed care is effective, so I think we are on the right track for hip fracture care……” (anaesthetist, 0302, conventional care)*
	Self-efficacy	Having self-confidence on capacity		*“We were trained several times, and I believe I can be proficient for this job.” (geriatrician, 0201, co-managed care)*
	Individual Stage of Change	Having a willingness to become more qualified		*“I am a novice, and I was transferred to the orthogeriatric ward last month, so I am worried about my work…… but I have a strong self-confidence to be better.” (nurse, 0501, co-managed care)*
**Process**	Opinion Leaders	Hospital president’s supervision		*“I am a decision-maker to address any issues, for example, the problems happened during the co-managed care delivery……” (administrator, 0601, co-managed care)*
	Pointed Implementation Leaders	Orthopaedic surgeons and geriatricians’ coordination		*“Several geriatricians and I jointly manage these patients and coordinate the care delivery within the multidisciplinary team…… I will report some issues to the hospital president if I cannot solve them.” (orthopaedic surgeon, 0101, co-managed care)*
	Planning		Insufficient quality control for the co-managed care	*“That would be better if we improve the quality assessment for the intervention, that will be prioritised to put in the to-do-list……” (orthopaedic surgeon, 0101, co-managed care)*
	Reflecting and Evaluating		Poor understanding about the effectiveness of the co-managed care	*“Actually, we don’t know the effectiveness of the intervention. Nobody can tell us. I think it would be helpful to our work if an annual report could be provided for us.” (geriatrician, 0201, co-managed care)*

*: identified different enablers and barriers through the cross comparison of co-managed care and conventional care groups

### Intervention characteristics

Compared to conventional care, almost all health providers considered the co-managed model of care as best practice for hip fracture management. This was primarily because the co-managed model of care provided expedited surgery and integrated management for older hip fracture patients.

However, all health providers perceived that the complexity of developing and implementing of the co-managed care was a challenge. They regarded that this type of model of care might not be easily adopted in hospitals where the infrastructure and clinicians were insufficient. Most participants thought the current paper-based checklist for implementing the co-managed care could be replaced by a digital device to enhance the work efficiency.

### Outer setting

#### National level

A guideline for hip fracture care to guide the establishment, implementation, and promotion of the co-management hip fracture care at the national level are lacking.

#### Hospital level

Participants regarded that the hospital president’s awareness of the importance of hip fracture management was considered an essential component. They should have a strong willingness to proactively prompt the establishment of the co-managed care, consisting of developing the orthogeriatric ward, prioritising the assessments and treatments for hip fracture patients, as well as offering incentives for health providers in the hospital. But some health providers mentioned that they had a lack of support to change the care model from the hospital level.

Additionally, all orthopaedic surgeons stated that they had increasing willingness to improve hip fracture management in China after they read more high-quality international publications and attended more international conferences around the implementation of the co-managed model of care for older patients with hip fracture.

There was a lack of international collaboration between Chinese hospitals and foreign hospitals for hip fracture care, which could have a negative impact on the development of the co-managed model of care.

### Inner setting

With the support of hospital presidents, laboratories and imaging departments prioritised hip fracture patients to complete preoperative assessment quickly, which helped accelerate the time to surgery. However, three geriatricians emphasised that lack of geriatricians was an obstacle for the promotion of the co-managed model of care, which was a tension for change. Beds in the orthogeriatric ward were still limited, which have a negative impact on the potential for expedited surgery.

As the multidisciplinary team leader, orthopaedic surgeons regularly updated the knowledge base of hip fracture care and organised group discussions with team members, which enabled improvement in the efficacy of work. All health providers indicated the co-managed model of care emphasised more frequent interactions and active collaborations between providers, which emphasised the need to thoroughly reorient the conventional care model, if the co-managed model of care were to be scaled up.

The lack of official software for communication and data sharing was a barrier. Staff reported concern about using “Wechat”, a public social media software, to interact and share patients’ data between health providers. It was very convenient for providers to share the patients’ information and communicate their conditions during the management. On the other hand, it was felt that use of the software might have a potential risk of private information leakage.

### Characteristics of individuals

A majority of health providers were enthusiastic, interested and confident about the intervention and had sufficient beliefs and knowledge on the success of intervention. They were also confident of their own capacity during the co-managed care delivery. Some novices, especially nurses in the early stage of their careers, worried about if they were sufficiently trained for their responsibilities, but they had strong willingness to sharpen their skills.

### Process

All stakeholders stated that hospital presidents should be viewed as the policy driver to urge and support the establishment of the co-managed model of care, as well as to supervise the care delivery, whereas orthopaedic surgeons and geriatricians were essential coordinators to facilitate the implementation of the co-managed care.

The lack of an annual report for the intervention evaluation and quality monitoring was identifiable barrier, which made the performance of the care model unknown.

### A conceptual framework for promotion strategies

**Fig. 1:**
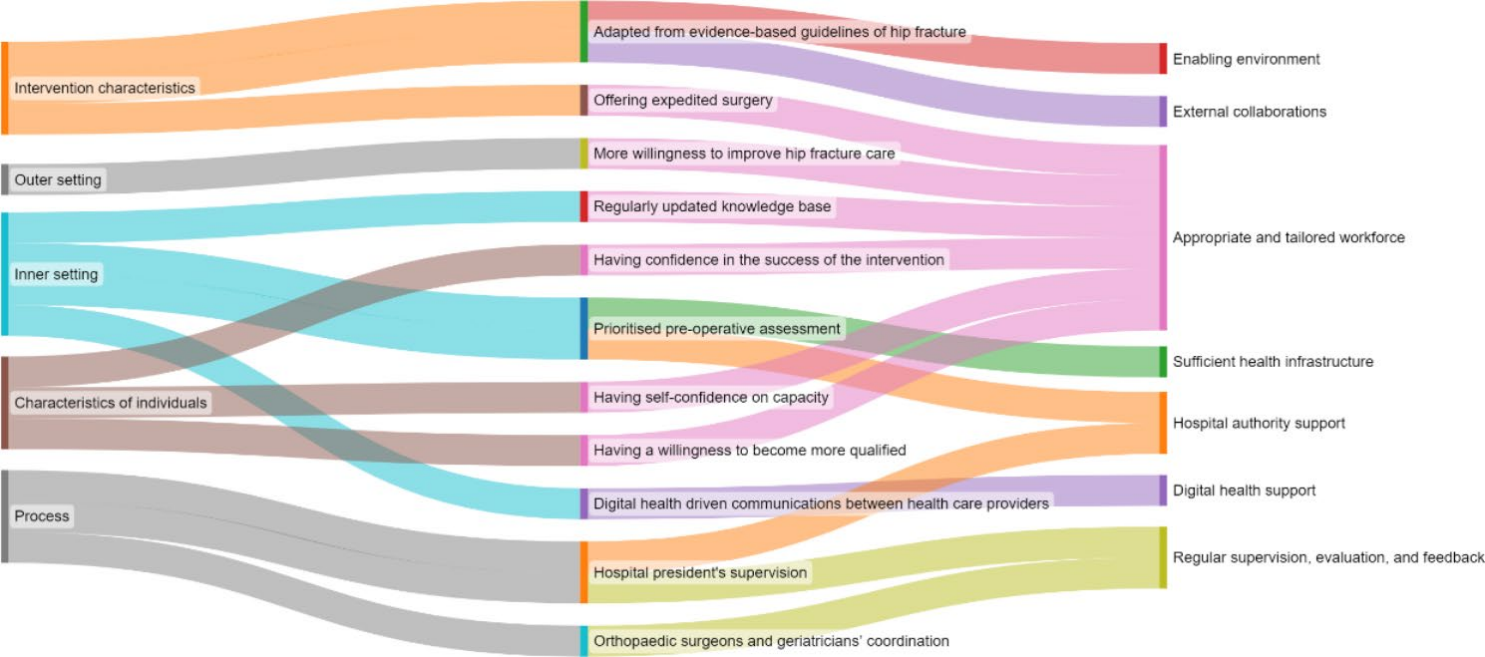
Identified barriers associated with seven essential components of implementation of the co-managed model of care

**Fig. 2:**
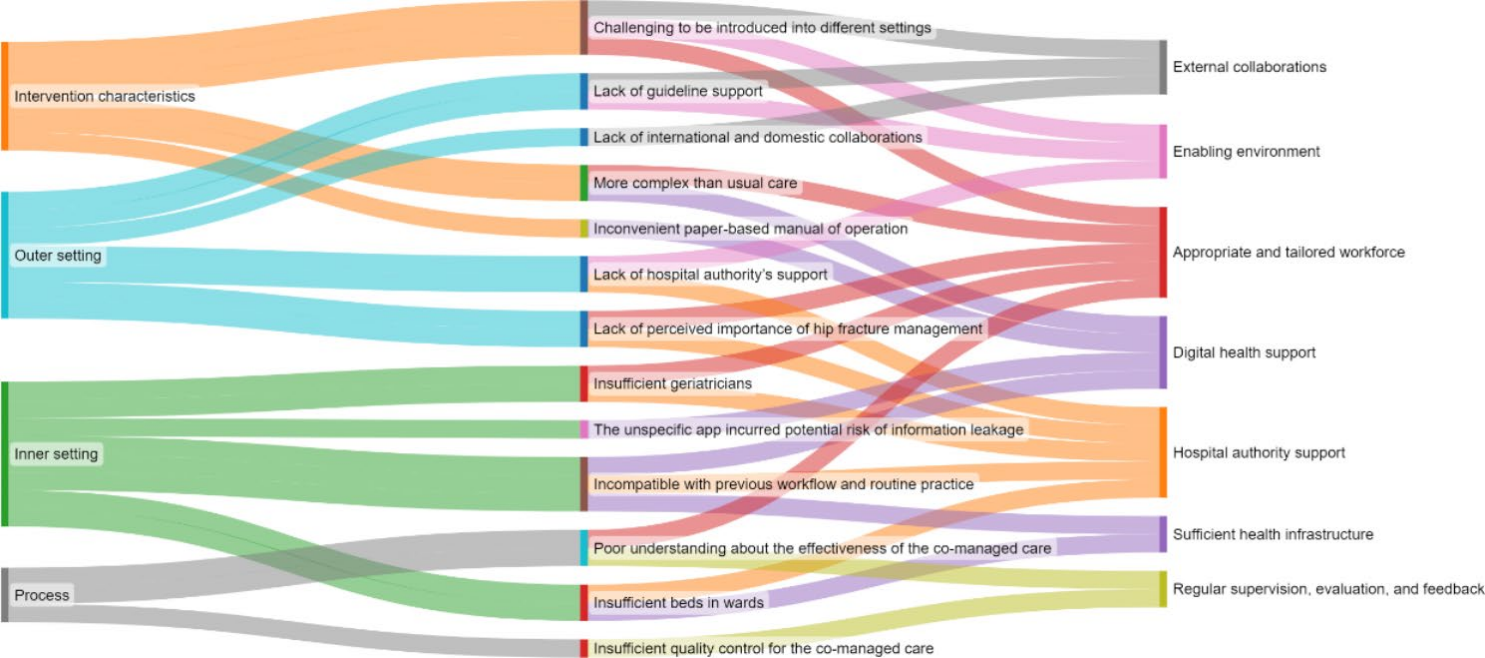
Identified enablers associated with seven essential components of implementation of the co-managed model of care

The strategies, associated with the CFIR constructs identified through the identification of enablers and barriers in the qualitative analysis, were inductively synthesised and developed into a conceptual framework, including three tiers with seven essential components, to inform the implementation and promotion of the hip fracture co-managed model of care in other hospitals (Figs. 1 and 2).

Tier 1: Hospital authority support is a key facilitator to dedicate the uptake of the co-managed model of care within hospitals. A national guideline of hip fracture management is another key facilitator that can build up an enabling environment for the co-managed model of care to ensure successful implementation.

Tier 2: Appropriate and tailored health workforce and sufficient health infrastructure are two fundamental components. Health providers should be qualified and actively engaged through offering regular training, using a unified and understandable protocol, and providing sufficient incentives. Also, engagement of health providers should be tailored according to setting and disciplines available. The hospitals with insufficient geriatricians, for example, could consider other disciplines as alternatives to integrate with orthopaedic surgeons. Health infrastructure is required to improve prior to the implementation of the co-managed model of care, including adequate beds and the availability of theatre schedule, for instance.

Tier 3: External collaborations, digital health support, and regular supervision, evaluation and feedback, are three important top-up components, which could enhance the feasibility, acceptability, and efficiency of the co-managed model of care. External collaborations promote the utilisation and engagement of domestic and international resources and partners. Digital health support facilitates the interaction between health providers and integrates the internal resources within the co-managed model of care. Regular supervision, evaluation and feedback improve quality of hip fracture care delivery, resulting from the lack of performance quality tracking and assessment.

**Fig. 3:**
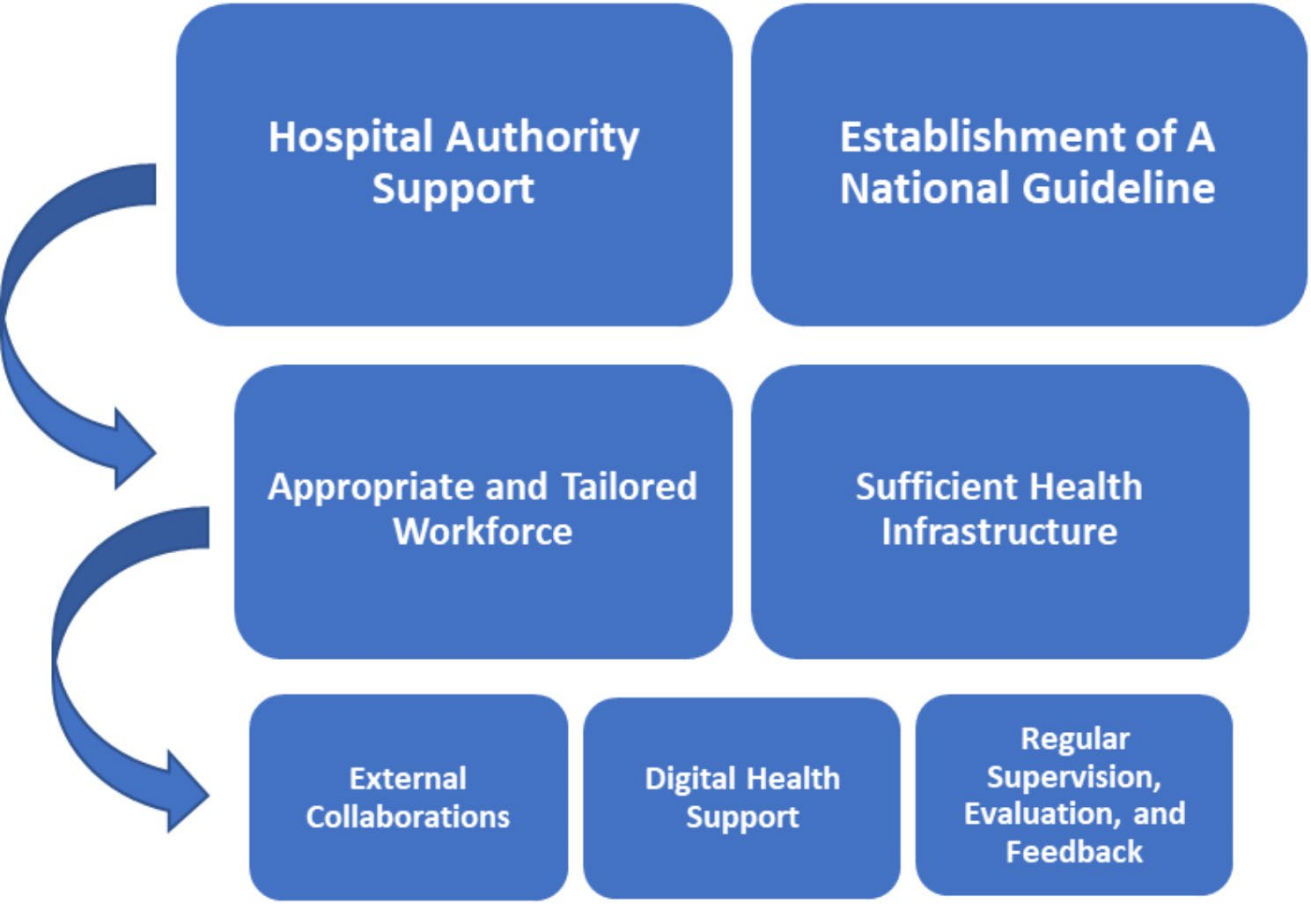
A conceptual framework for the implementation and promotion of the hip fracture co-managed model of care

The three tiers with the seven components are important to improve the implementation of co-managed model of care from different perspectives. The tier 1 and 2 facilitators are necessary to be achieved if this care model would be well-organised, while the tier 3 factors would contribute the high quality and efficiency to the implementation of this care model (Fig. 3).

## Discussion

The aim of the study was to use the CFIR framework to delineate the critical enablers and barriers that influence the successful implementation of co-managed model of care and to create a conceptual framework for scale up of this care model. The results of these CFIR-inspired interviews clarified that essential components of implementation for this care model must be addressed if the co-managed model of care is to be successfully integrated into hip fracture care in China.

During the delivery of co-managed care, the primary concern was the lack of hospital authorities’ willingness and resolution to change the conventional care model. Without hospital authority engagement, innovation of any model of care is impossible in hospital. Lemon and Uchiyama emphasised that leadership engagement was the key to a proposed intervention’s promotion [19, 20]. More evidence, related to the burden of hip fracture and effective interventions, needs to be disseminated to the hospital administrators to seek prioritisation of resource allocation within the hospital [6, 21, 22]. Creating an enabling environment is vital to streamline hip fracture care delivery in a smooth, high-quality, and efficient manner. That is, the publication of a Chinese guideline of hip fracture management endorsed by governments might be a solution that urges hospital presidents to change their mindset for the care model transformation.

There was also another concern about whether the new care model was applicable to other hospitals. Despite the effectiveness, this multicomponent intervention was relatively more complex, involving various disciplines each spending more time in practice, compared to the conventional care. As a result, this could be a major challenge in resource-constrained hospitals, with a lack of human resources and infrastructure. The availability of health care professionals was seen as a key factor for the successful implementation of the intervention [23]. In contrast with western countries, geriatric medicine is relatively underdeveloped in China, with only one quarter of hospitals above the level of primary health facilities in Beijing being equipped with a geriatric department [24]. As such, some studies have stated that an appropriate alternative solution could be to locally assign a tailored intervention implementer for their own contexts, particularly in resource-limited settings [25, 26]. For example, leveraging task-shifting approaches to replace geriatricians with other disciplines to address comorbidities of patients for earlier surgery might be feasible. Furthermore, the capability and skills of the health workforce are the other two important elements which can have an impact on health outcomes and health service coverage [27]. A regular training program with frequent experience sharing among health providers could be implemented, which was an approach previously verified as an effective solution in sharpening skills of health providers [28]. An understandable protocol with a streamlined workflow being circulated within an operational team might make it easy to implement a complex intervention, with potential to bridge the gap of capacity building for new staff in resource-limited areas [29]. Financial incentives were also seen as an important solution to trigger the motivation of health providers, so that they were willing to actively adapt the environment and culture in the multidisciplinary team, skill-up, and ensure the care model operated well. A previous study has shown that monetary compensation for health providers depending on their performance facilitated the uptake of intervention, particularly in the early stage of the implementation [25]. Furthermore, in order to improve the quality of care, the Pay-for-Performance model can also be considered. That means a financial incentive is paid to health providers for achieving a quality-related patient target [30]. An early study conducted in the US and England has shown that Pay-for-Performance led to significant enhancements in health outcomes of patients with hip fracture [31].

In addition to endeavours of health providers, the necessary infrastructure must be sufficiently provided to support care delivery [32]. For instance, a fast-track mechanism inside hospitals could be established to ensure the availability of medical resources, such as expedited biochemical testing and imaging diagnosis, to allow patients to be admitted to the ward quickly. Moreover, the size of the ward should be sufficiently large to tackle the increasing number of patients, so as to reduce patients’ delays during transferring from the emergency department to the ward. The use of theatre should be extended to reduce the time to surgery from admission as well. These proposed recommendations for the availability of health infrastructure could therefore enable the co-managed model of care to smoothly implement, which are aligned with the surgery strategies in Low- and Middle-Income Countries, advocated by The Lancet Commission in 2015; that is, government’s funding should be primarily invested in the establishment of health infrastructure [33]. Despite challenges, with the endorsement of hospital authorities and governments around the uptake of the co-managed model of care, insufficiency of geriatricians and health infrastructure should be resolved in the future. These four components make it more possible to implement the co-managed model of care in other hospitals if the tier 1 and 2 facilitators would be well-achieved.

Additionally, there are three additional top-up components to potentially enhance the implementation of the co-managed model of care. A decision support system, facilitated by digital health technology, might be an efficient way to facilitate the implementation of a complex intervention across various settings and improve care delivery from the health system perspective, including digitalised data sharing, instant communications, referral coordination and activity scheduling [34, 35]. In order to improve the quality of care delivery, a well-established audit strategy has been previously demonstrated to improve protocol fidelity and health outcomes [36]. For quantifying and standardising the audit strategy, a set of pre-defined indicators that address implementation quality of hip fracture care to improve the practice of health providers is required. In parallel, supervision activities should be established for the routine monitoring of the indicators. For example, a well-engaged opinion leader, such as an orthopaedic surgeon or a geriatrician, should play a role in auditing the operation of the care model. A systematic scoping review argued that the development of valid indicators with a regular supervision is important to ensure the success of an intervention [37]. Regular evaluation and feedback of the intervention allow health providers to understand the performance of the co-managed care, which can also improve their self-confidence and self-efficacy [38]. Collaborations across settings, areas, or countries are very important to enhance an intervention promotion, because there is very limited experience and underdeveloped conception of the hip fracture co-managed model of care in China. For instance, several high-income countries launched local chapters of the Global Fragility Fracture Network to share their experiences around hip fracture treatment and management [39]. China’s active engagement in this network might help the promotion of the co-managed model of care in the future. Finally, the development of a national guideline of hip fracture management is necessary to the implementation of the co-managed model of care. An audit conducted in UK argued that guideline driven integrated care delivery can significantly improve quality of hip fracture care and health outcomes through consistent delivery of the guideline [40].

There are two strengths to this study. First, a mix of participants were recruited, including those who were actively involved in implementing the co-managed model of care and those who were not. This enables ascertainment of more comprehensive insight into the barriers and enablers of the co-managed model of care from both sides. Second, the Consolidated Framework for Implementation Research was adopted. The framework uses a systematic approach that can help accurately identify all conceptual factors that influence the implementation of the intervention, enhancing the generalizability and interpretability of research findings. There are two limitations. First, the barriers and enablers identified in this study may not be transferable because of the limited sample size and its representativeness. Participants were from three tertiary hospitals in Beijing, a resource-rich area, so it is possible that all specific contextual factors which may impact implementation could not be identified. Second, the samples were more from the hospitals with the conventional care due to only one hospital with the co-managed care as the interventional group but five hospitals as the control group in that study we mentioned in the introduction. It may potentially influence the research findings, because control group participants were less familiar with the uptake of co-managed model of care, compared to their counterparts.

## Conclusions

Despite the complexity, the co-managed model of care could be introduced and promoted in the context of Chinese hospitals through primarily obtaining the hospital authority and government’s supports and secondarily providing sufficient experienced health workers and infrastructure. Digital health approaches, external collaboration, and strategies of audit and feedback would be likely top-up facilitators.

### Electronic supplementary material

Below is the link to the electronic supplementary material.

Supplementary Material 1

Supplementary Material 2

## Data Availability

The dataset is managed by Beijing Jishuitan Hospital. The data access request can contact the corresponding author.

## Abbreviations

COREQ: Consolidated Criteria for Reporting Qualitative Research
CFIR: Consolidated Framework for Implementation Research
